# A Humanin Derivative Reduces Amyloid Beta Accumulation and Ameliorates Memory Deficit in Triple Transgenic Mice

**DOI:** 10.1371/journal.pone.0016259

**Published:** 2011-01-17

**Authors:** Takako Niikura, Elkhansa Sidahmed, Chiho Hirata-Fukae, Paul S. Aisen, Yasuji Matsuoka

**Affiliations:** 1 Faculty of Health Sciences, Simon Fraser University, Burnaby, British Columbia, Canada; 2 Department of Neurology, Georgetown University, Washington, D. C., United States of America; 3 Department of Neurosciences, University of California San Diego, La Jolla, California, United States of America; Boston University School of Medicine, United States of America

## Abstract

Humanin (HN), a 24-residue peptide, was identified as a novel neuroprotective factor and shows anti-cell death activity against a wide spectrum of Alzheimer's disease (AD)-related cytotoxicities, including exposure to amyloid beta (Abeta), *in vitro*. We previously demonstrated that the injection of S14G-HN, a highly potent HN derivative, into brain ameliorated memory loss in an Abeta-injection mouse model. To fully understand HN's functions under AD-associated pathological conditions, we examined the effect of S14G-HN on triple transgenic mice harboring APP_swe_, tau_P310L_, and PS-1_M146V_ that show the age-dependent development of multiple pathologies relating to AD. After 3 months of intranasal treatment, behavioral analyses showed that S14G-HN ameliorated cognitive impairment in male mice. Moreover, ELISA and immunohistochemical analyses showed that Abeta levels in brains were markedly lower in S14G-HN-treated male and female mice than in vehicle control mice. We also found the expression level of neprilysin, an Abeta degrading enzyme, in the outer molecular layer of hippocampal formation was increased in S14G-HN-treated mouse brains. NEP activity was also elevated by S14G-HN treatment *in vitro*. These findings suggest that decreased Abeta level in these mice is at least partly attributed to S14G-HN-induced increase of neprilysin level. Although HN was identified as an anti-neuronal death factor, these results indicate that HN may also have a therapeutic effect on amyloid accumulation in AD.

## Introduction

Humanin (HN) is a multi-functional 24-residue peptide (amino acid sequence: MAPRGFSCLLLLTSEIDLPVKRRA) whose cDNA was isolated from an Alzheimer's disease (AD) patient's occipital lobe of brain, a region that remains generally intact in many AD cases [1] (reviews in [2], [3], [4]). HN suppresses the neuronal death caused by all AD-related insults so far tested *in vitro* including the key cytotoxic molecule in AD, amyloid beta (Abeta) 1-42 [1], [5], [6]. HN suppressed cell death caused by Abeta toxicity not only in primary neurons but also in cerebrovascular smooth muscle cells, a model of amyloid angiopathy [7]. HN is also effective against cell death caused by non-AD-related insults under different experimental settings, such as serum depriviation, prion peptide118-135, IGFBP3 (insulin-like growth factor binding protein 3), staurosporine etc. [8], [9], [10], [11]. However, HN is not effective against some insults such as etoposide, suggesting that HN is not a general anti-apoptotic agent.

It has been hypothesized that HN stimulates its receptor(s) and activates signaling cascade(s) to exert its effects [2], [4]. Upon HN stimulation, G protein coupled receptors, formyl peptide receptor-like (FPRL) 1 and FPRL2 [12], [13], induce increase of Ca^2+^ flux and activation of extracellular signal-regulated kinase (ERK), while a receptor complex consisting of gp130, CNTFR, and WSX-1 [14] induces activation of a transcription factor, signal transducer and activator of transcription 3 (STAT3). In addition, three receptor-independent mechanisms have been proposed. (I) Intracellular HN bound to pro-apoptotic Bcl-2 family members, Bax, BimEL, and tBid, and blocked cytochrome c release from mitochondria, leading to inhibition of apoptosis [11], [15], [16]. (II) HN increased cellular ATP levels in human lymphocytes and a muscular cell line [8], [17], [18], [19], [20]. (III) Extracellularly added HN was detected in the cells and suppressed apoptosis induced by IGFBP3 [10].

Through structure-function analyses, we found that a substitution of Gly for 14th Ser (S14G-HN) increased potency 1000-fold [1]. S14G-HN ameliorated amnesia caused by muscarinic receptor antagonists [21], [22], [23] and Abeta in mice [23], [24]. S14G-HN also ameliorated symptoms and/or pathology in rodent stroke model [25], [26] and diabetes models [27], [28]. These findings suggest the potential of HN for therapeutic application in AD and other diseases.

To evaluate the effect of HN derivatives *in vivo*, Abeta injection model was used in the previous studies [23], [29], [30]. In this model, Abeta administration induces amnesia in rodents, decreases the number of cholinergic neurons [31], and reduces choline acetyltransferase activity [32], [33], [34], [35] Although the Abeta injection model is a simple and convenient model, the transgenic mouse model has some advantages, for instance production of multiple Abeta species through physiological process and amyloid plaque formation. The triple transgenic mice harboring APP_swe_, tau_P310L_, and PS-1_M146V_ (3xTg-AD) [36] have the advantage of the age-dependent development of multiple pathological events relating to AD. In these mice, memory impairment was observed by Morris water maze test at 9 months of age. Soluble Abeta was observed from 9 months of age and plaques from 14 months by immunohistochemical analyses. They also develop tau pathology: total tau protein increased age-dependently, correlating with a decrease of soluble tau and an increase of insoluble tau [37]. Therefore, they are beneficial to examine the effect of therapeutic candidates on pathological changes under the complex physiological conditions.

We previously demonstrated that Abeta-induced amnesia was suppressed by intracerebroventricular injection of S14G-HN [23]. To fully understand HN's functions on complex AD-relevant pathology, in this study, we used triple transgenic mouse model. We found that S14G-HN ameliorated cognitive function of 3xTg-AD mice and reduced amyloid burden accompanied by an increase in neprilysin level in the hippocampal region.

## Materials and Methods

### Animals and treatment

Triple transgenic (3xTg-AD) mice were housed under 12 hour light, 12 hour dark cycles with food and water provided ad libitum. Male and female 3xTg-AD mice (n = 9 in each group) at the age of 13 months (13.2±0.6) were treated intranasally with 10 nmol of S14G-HN (Peptide Institute, Japan) or vehicle (water) for 3 and half months. Treatment was carried out 5 days a week (weekdays) for the initial 2.5 months and everyday during the last 1 month. After 3 months of treatment, 7 male and 9 female mice in the vehicle control group and 9 male and 7 female mice in the S14G-HN treatment group were subjected to behavioral analyses. After 1 week of the last behavioral test (24 hours after the last treatment), mice at 17 months of age were sacrificed by cervical dislocation as described in Planel et al. [38]. Half hemispheres of the brains were rapidly frozen for immunoblot and ELISA analyses and the other half hemispheres were fixed with 4% paraformaldehyde for histological analyses. To examine the delivery of S14G-HN to brain, 3xTg-AD mice (female, 20 months of age) were treated intranasally with 10 nmol of S14G-HN or vehicle (water). After 1 hour of administration, mice were sacrificed and brains were fixed with 4% paraformaldehyde for histological analyses. All experimental procedures were approved by the Georgetown University Animal Care and Use Committee (Protocol #07-016).

### Open Field test

The open field test was performed as described in Takahashi et al. [39]. The apparatus was a rectangle container (45 cm ×35 cm and 15 cm height). The peripheral area was designated within 10 cm from the walls. Each mouse was placed at a corner and the activities were recorded for 5 min. Mouse behaviors (walking distance, speed, and duration spent in peripheral area) were analyzed by TopScan software (Cleaver System Inc., VA).

### Novel object recognition task

The novel object was performed as described in Nagai et al. [40]. The apparatus was a rodent cage (43 cm ×27 cm ×22 cm). Two identical objects were presented to a mouse in the apparatus for 5 min and the time spent exploring the objects was recorded. After 30 min, the mouse was returned to the arena where one object was replaced with a novel object and the mouse was allowed to explore for 5 min. The exploring time for each object was recorded. All behavior was recorded and analyzed by TopScan software. The object discrimination of normal rats is greatest in the initial two minutes [41], so we focused on the first two minutes of exploring time to determine cognitive function of mice and calculated Discrimination ratio (DR) as described in Winters et al. [42]: DR  =  (N-F)/T, where N is time spent exploring the novel object, F is time spent exploring the familiar object, and T is total time spent exploring two objects.

### Morris water maze

The Morris water maze was performed as described by Matsuoka et al. [43]. The circular pool (diameter 150 cm, height 30 cm, painted white) was filled with water (22±1.0°C). A circular, white escape platform (diameter 10 cm) was submerged 1 cm below the water surface, 30 cm off the rim of the pool. Training trials, 120 sec each, were performed twice a day during 5 consecutive days. The platform location and the animal's starting point were held constant within each pair of daily tests, but the location of the platform and the animal's starting point were rotated by 90° every day. The mice were allowed to stay on the platform for 20 sec before and after each trial. On the fifth day, a probe test was preformed after the second daily trial. The platform was removed from the maze and swimming behavior was recorded for 2 min. Mice were then subjected to a visible platform test. A black escape platform was placed at 1cm above the water in the same place in training trials in the 5th day. Each mouse was released into the water and the time taken to find the platform was recorded (maximum 60 seconds). Animals that did not climb on the platform during this test were excluded from the statistical analysis. All behavior was recorded and analyzed by TopScan software.

### Abeta ELISA

The method is described in Hirata-Fukae et al. [37]. Briefly, brains were homogenized in buffer (50 mM Tris-HCl, pH 7.4, 1 mM EDTA) with protease inhibitors (Halt Protease Inhibitor Cocktail, Pierce) and phosphatase inhibitors (Phosphatase inhibitor Cocktail set, Calbiochem). Soluble Abeta was extracted with 0.4% diethylamine as described in Schmidt et al. [44] and subjected to ELISA as described in Horikoshi et al. [45].

### Immunohistochemical analysis

Immunohistochemical staining was described in Hirata-Fukae et al. [37]. Briefly, 30 µm sagittal sections were subjected to immunostaining using anti-HN (P04) [46], anti-Abeta antibody (82E1, IBL), anti-phosphorylated tau (Ser202) (AT8, Pierce), anti-phosphorylated tau (Thr231) (AT180, Pierce), or anti-insulin degrading enzyme (IDE) (Abcam) and biotin-conjugated secondary antibody followed by visualization with avidin-fluorescein for HN or ABC method (Vector Laboratories) for others. Immunostaining with anti-neprilysin (NEP) antibody (clone 56C6, Abcam) and anti-synaptophysin (SVP-38, Sigma) were performed as described in Iwata et al. [47] and Tomiyama et al. [48], respectively. Immunostaining with anti-phosphoTyr705-STAT3 antibody (Cell Signaling) was performed according to the manufacturer's instruction using anti-rabbit-DyLight488 secondary antibody (Pierce). For quantitative analysis, staining intensity was measured with ImageJ.

### Immunoblot analysis

Brain homogenate was subjected to SDS-PAGE (Nu-PAGE, Invitrogen), followed by transfer to polyvinilydendifluoride membrane (Millipore). Membranes were blocked with 5% BSA in TBS-T (20 mM Tris-HCl pH 7.6, 136 mM NaCl, and 0.1% Tween 20), and incubated with primary antibody and secondary antibody (horseradish peroxidase conjugated anti-mouse or rabbit IgG) in 2% BSA in TBS-T, followed by detection with enhanced chemiluminescence reagent (Thermo Scientific). Primary antibodies were anti-APP (IBL), anti-sAPPalpha (IBL), anti-phosphorylated-tau (Ser202) (AT8, Pierce), anti-phosphorylated tau (Thr231) (AT180, Pierce), anti-total tau (Tau46, Sigma), and anti-beta-actin (Sigma). Anti-phosphoTyr705-STAT3, anti-total STAT3, anti-phosphoSer473-Akt, anti-phosphoThr308-Akt, and anti-total Akt were from Cell Signaling.

### NEP activity assay

F11 neuronal cells were cultured in Ham's F12 medium supplemented with 18% fetal bovine serum, penicillin/streptomycin (Gibco). Cells were treated with 500 nM S14G-HN or vehicle (water) for 2 hours. Cell lysate was subjected to NEP activity assay as described in Hemming et al. [49]. Briefly, cell lysate was mixed with 1 mM captopril (MP Biomedicals) and incubated with substrate, 3-dansyl-D-Ala-Gly-p-(nitro)-Phe-Gly (DAGNPG; Bachem) in the presence or absence of 10 mM phosphoramidon (American peptide). Reaction was stopped by heating. After clarifying reaction mix by centrifugation, fluorescent intensity was measured by a plate reader (excitation 340, emission 535, TECAN ULTRA).

### Data analysis

Data was statistically analyzed by t-test, Mann-Whitney U test, (SPSS) or non-parametric ANOVA (Kruskal-Wallis test) followed by Dunn's post-hoc test (Prism).

## Results

### S14G-HN does not affect locomotor activity of 3xTg-AD mice

**Figure 1:**
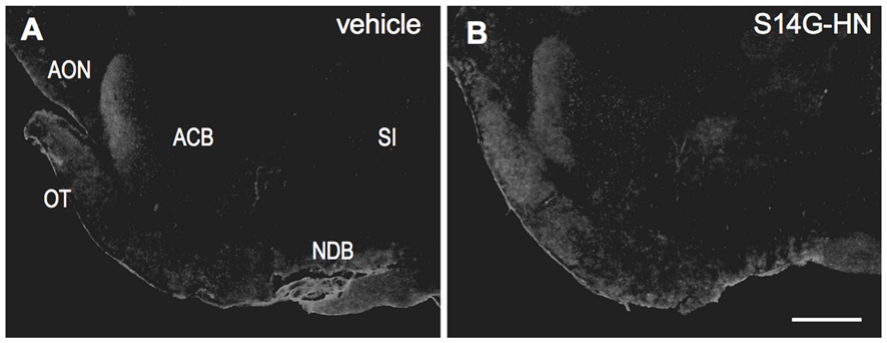
Delivery of S14G-HN to brain by intranasal administration. 3xTg-AD mice (13 month-old) were intranasally administrated with S14G-HN (B) or vehicle (A), and sacrificed after 1 hour of administration. Brains were fixed with 4% paraformaldehyde and 30 µm sagittal sections of brains were subjected to immunostaining using anti-HN antibody (P04) and biotin-conjugated secondary antibody followed by visualization with avidin-fluorescein. ACB, Nucleus accumbens; AON, Anterior olfactory nucleus; NBD, Diagnal band nucleus; OT, Olfactory tubercle; SI, Substantia innominata. Bar = 500 µm.

Intranasal administration is a method for delivery of therapeutic agents directly and efficiently to the brain [50]. We treated 3xTg-AD mice with S14G-HN intranasally, and performed immunohistological staining with anti-HN antibody (P04) [46]. Based on the study on IGF-I [51] we focused on olfactory tract area, and found positive immunostaining in olfactory tubercle region (Fig. 1). This suggests that S14G-HN is transported into brain through a pathway associated with the peripheral olfactory system as described in [51].

**Figure 2:**
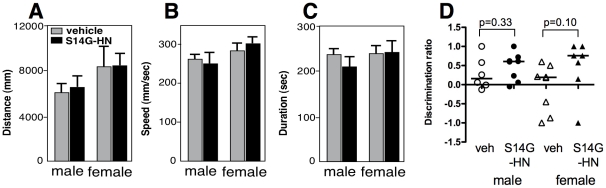
The effect of S14G-HN on locomotor activity of 3xTg-AD mice. Male and female 3xTg-AD mice were subjected to behavioral analyses, Open field test (**A–C**) and Novel object recognition task (**D**), after 3 months intranasal administration of S14G-HN or vehicle. In Open field test, walking distance (**A**), walking speed (**B**) and duration stayed in the peripheral region (**C**) were measured. No significant difference was observed between S14G-HN and vehicle-treated mice in both genders. In Novel object recognition task, time of sniffing behavior to two objects was measured. In the first trial, no preference was observed in sniffing time of two objects (data not shown). The discrimination ratio in the second trial is shown (**D**). Bars indicate median, and p =  indicates p value of Mann-Whitney U test. Number of animals are male vehicle control: n = 7, S14G-HN: n = 9, female vehicle control: n = 8, S14G-HN: n = 7.

To examine the effect of HN, we designed a 3-month treatment study using male and female 3xTg-AD mice (n = 9 in each group) at the early plaque-bearing stage. Since the progression of Abeta pathology in our colony is slower than that in the original colony [37], we started the treatment of mice at the age of 13 months (13.2±0.6). S14G-HN showed complete neuroprotective activity at 1nM of concentration *in vitro* [1], [6]. To achieve approximately 1nM of concentration in CNS, we chose a dose of S14G-HN at 10 nmol/day, based on the finding that intranasal administration of 5 nmol [^125^I]-IGF-I resulted in 0.32–1.44 nM of concentration in CNS region [51]. We treated male and female 3xTg-AD mice intranasally for three months with 10 nmol S14G-HN. After three months of treatment, we performed behavioral tests. In the Open Field test (Fig. 2A–C), no significant difference was observed between the vehicle and S14G-HN treated mice in total moving distance (Fig.2A), walking speed (Fig.2B), and time spent in peripheral area (Fig.2C). These results indicate that S14G-HN did not affect anxiety-induced locomotor activity and exploratory behaviors. In the Novel object recognition test (Fig. 2D), S14G-HN treated mice showed a trend toward improved recognition, though not reaching statistical significance.

### S14G-HN ameliorates deficit in spatial learning and memory in 3xTg-AD mice

**Figure 3:**
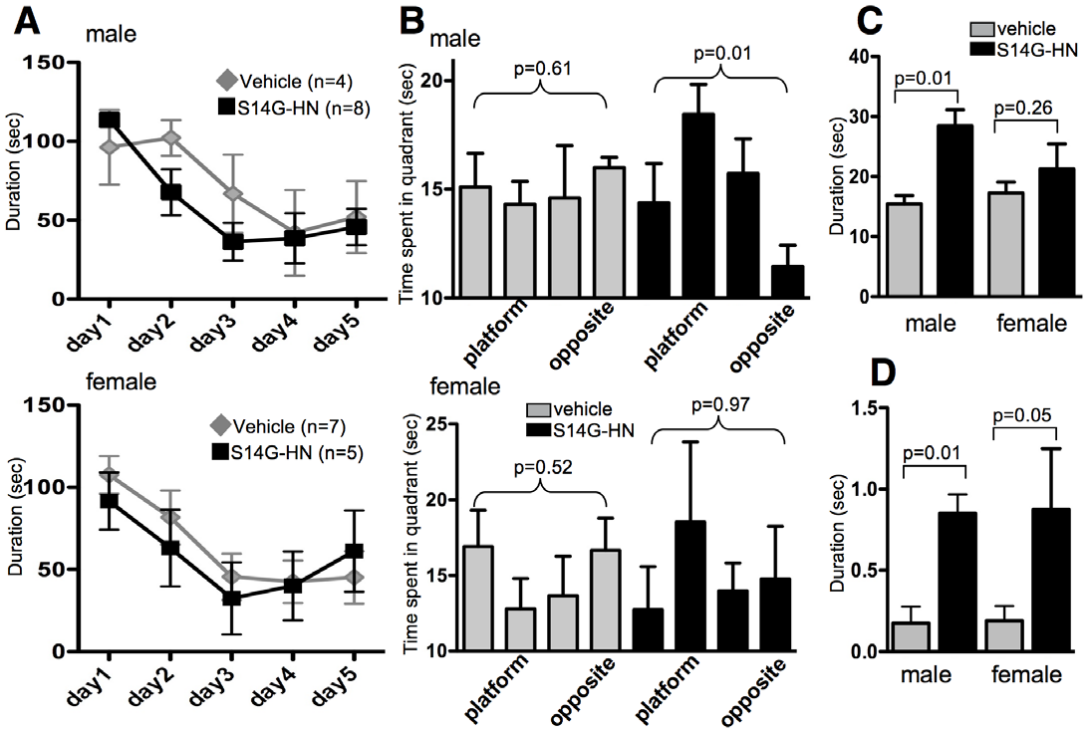
The effect of S14G-HN on spatial memory in 3xTg-AD mice. Male and female 3xTg-AD mice were subjected to Morris Water Maze after 3 months of intranasal administration with S14G-HN or vehicle. **A.** Training sessions. Training trials (120 sec each) were performed twice a day for 5 days. Time spent to find platform in first trial in each day is shown as mean±SEM. **B–D.** Probe test. Time spent in each quadrant is shown as mean±SEM (**B**). Mice which did not find the visual platform were considered abnormal mice and data of these mice was excluded from analysis (vehicle male = 3, female = 2, S14G-HN male = 1, female = 2). P values of non-parametric ANOVA test (Kruskal-Wallis test) are shown. Time spent within 60 cm from the platform region during first 60 seconds of probe test is shown as mean±SEM (**C**). Time spent within platform area during first 30 seconds of probe test is shown as mean±SEM (**D**). In C and D, p =  indicates p values of Mann-Whitney U test.

In the Morris water maze test, training trials (2 min each) were performed twice a day for 5 days. To assess flexible learning ability, the position of the platform was rotated everyday. The parameter of learning flexibility was analyzed by looking at the block of first trials performed each day (Fig. 3A). S14G-HN treated male mice showed a trend of shorter latency than control on the second day. In female mice, no significant difference was observed between S14G-HN-treated and control mice. On the fifth day, the probe test was performed after the second daily trial. Time spent in each quadrant of pool was significantly different in S14G-HN treated male mice but not in vehicle control male mice by non-parametric ANOVA (Fig. 3B). Post-hoc test on values of S14G-HN-treated male mice detected a significant difference between platform quadrant and opposite quadrant (p<0.05). Consistently, time spent in the area within 60 cm from the platform location was significantly longer for S14G-HN treated male mice than for vehicle control mice (Fig. 3C). These results show that S14G-HN-treated male mice stayed close to the platform location longer than vehicle control mice, suggesting better cognitive and memory function of S14G-HN-treated mice than control. We also measured the duration that mice spent in the platform location (Fig. 3D). In the first 30 sec of probe test, S14G-HN-treated male mice stayed in the platform area significantly longer than control male mice. S14G-HN treated female mice showed a trend similar to the male mice, though the difference was not significant (Fig. 3B–D). Swimming speeds of S14G-HN treated and control mice were virtually the same in probe test (data not shown), indicating that locomotion activity was not affected by S14G-HN.

### Abeta level was reduced in brain of S14G-HN-treated 3xTg-AD mice

**Figure 4:**
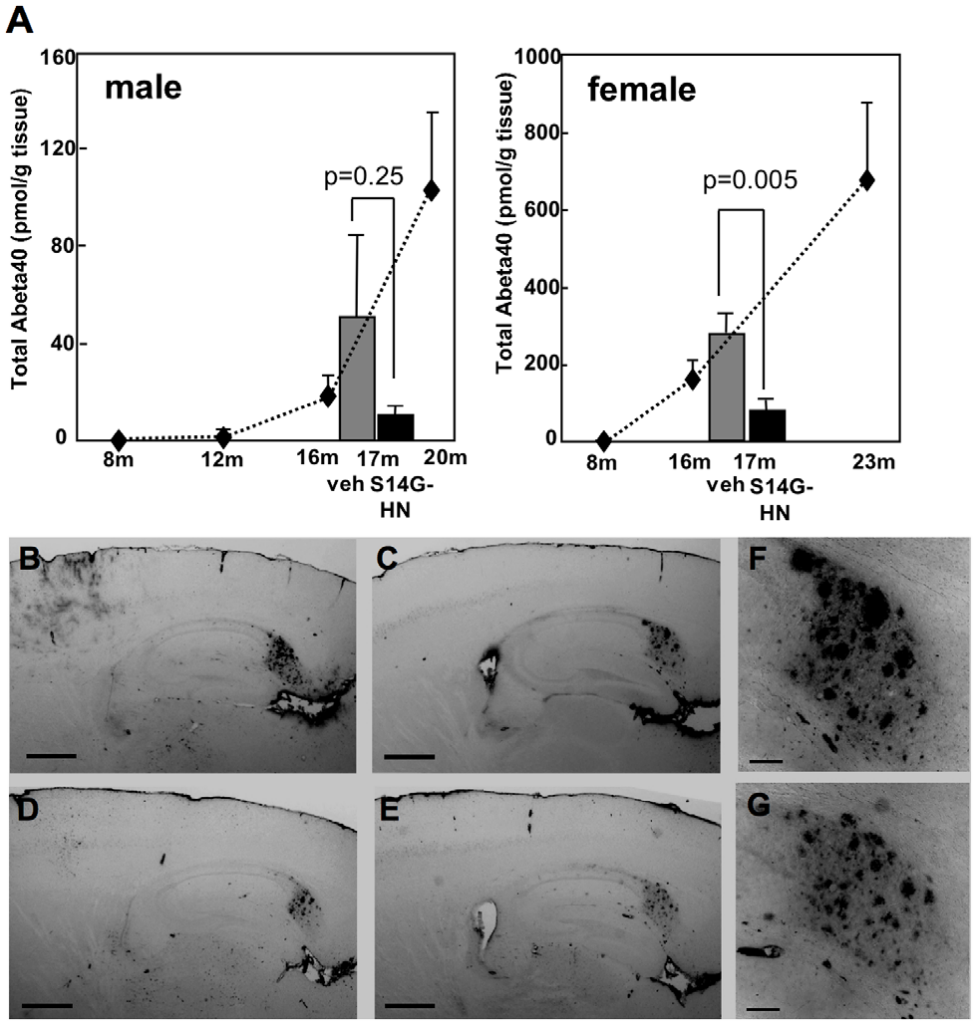
Brain Abeta level was sustained in S14G-HN-treated 3xTg-AD mice. After behavioral tests, mice were sacrificed at 17 months of age. Abeta was extracted from brain homogenates using formic acid. **A**. Amount of human Abeta40 was measured by ELISA and shown as mean±SEM. P values of Student t-test are shown. Number of animals were vehicle male = 7, female = 9, S14G-HN male = 9, female = 7. Lower level of Abeta was detected in S14G-HN treated mice (black columns). Statistical significance was observed in female but not male compared with vehicle treated mice (dark grey columns). Dotted line plots show Abeta40 levels of naïve 3xTg-AD mice at the indicated age [37]. **B–G**. Immunohistochemical analysis of amyloid plaques. Brains were fixed with 4% paraformaldehyde and 30 µm sagittal sections were subjected to immunostaining using anti-Abeta antibody (82E1) and biotin-conjugated secondary antibody followed by visualization with ABC method. **B, D, F.** vehicle-treated female mice, **C, E, G.** S14G-HN-treated female mice. **F, G.** magnified view of subculum regions in C and E, respectively. Bar  = 500 µm in B-E,  = 100 µm in F, G.

Since Abeta pathology may correlate to cognitive function, we examined the levels of Abeta in brain from 3xTg-AD mice treated with S14G-HN or vehicle by ELISA. Total Abeta40 in vehicle control, both male and female (Fig. 4A grey columns), showed levels consistent with the expected age-dependent increase of Abeta in naïve mice reported in Hirata-Fukae et al. (2008) (Fig. 4A dotted lines). Compared with vehicle control, the levels of Abeta40 in S14G-HN mice (Fig. 4A black columns) were markedly low in both male and female mice, though statistical significance was detected only in females. A similar trend was observed in soluble Abeta40 levels in both genders, though the difference was not significant (data not shown). We also performed ELISA to detect total Abeta42. Since Abeta42 level is still low at this age of 3xTg-AD mice, many samples resulted in undetectable level: 2 out of 7 vehicle male, 6 out of 9 S14G-HN-treated male, none of vehicle female, and 5 out of 7 S14G-HN-treated female mice showed undetectable level of Abeta42. Given zero as a value of these undetectable samples, Abeta42 levels (mean±SE, pmol/g tissue) were vehicle male (n = 7); 6.5±3.9, S14G-HN male (n = 9); 3.8±2.1, vehicle female (n = 9); 46.0±20.4, S14G-HN female (n = 7); 10.4±6.7. P values of t-test were 0.54 and 0.007 for male and female, respectively. These results indicate that Abeta42 level had a trend similar to that of Abeta40 (Fig. 4), i.e. decreased level of Abeta in S14G-HN-treated mice.

To confirm the finding obtained by ELISA, we examined the amount of amyloid plaques by immunohistochemical analysis using anti-Abeta antibody 82E1, which specifically recognizes Abeta but not APP. Representative results are shown in Fig. 4B–G. We stained six sections per mouse at 150 µm intervals and evaluated the number of plaques (count), total area, and integrated density of staining in subiculum region of each section (summarized in Table 1). Both total area and integrated density were significantly lower in S14G-HN-treated 3xTg-AD female mice than the vehicle control. The similar trend was observed in male mice as in female. As observed in the results of ELISA (Fig. 4A), no statistical significance was detected in males because of large individual variations (data not shown). Combined with the results of ELISA, these results indicate that S14G-HN suppresses the age-related increase of plaque burden in 3xTg-AD mouse brains.

**Table 1:** Decreased amyloid burden in S14G-HN-treated mice.

		Count	Total Area	Average Size	IntDen
Vehicle	Mean	259.8	74364	165.1	16048999
	SD	37.5	14899	19.1	4509234
S14G-HN	Mean	208.5	35807	56.6	7610845
	SD	54.1	13482	0.02	2787706
% of veh	Mean	80.3	48.2	34.3	47.4
	SD	20.8	18.1	0.01	17.4
t-test	P =	0.176	0.009	0.016	0.024

Brain sections were prepared as described in Fig. 4. Subiculum region of each section was subjected to quantitative analysis using ImageJ. Number of plaques (Count), total immunopositive region (Total Area), and integrated density (IntDen) were measured and Average size of particles (plaques) was calculated (Total Area/Count). Value of six sections of each animal at 150 µm intervals were summed. Mean and SD of four female animals per group are shown. The relative ratio of S14G-HN-treated group to vehicle group is indicated as % of vehicle. P =  indicates P value of t-test.

### APP production and processing was not affected by S14G-HN

**Figure 5:**
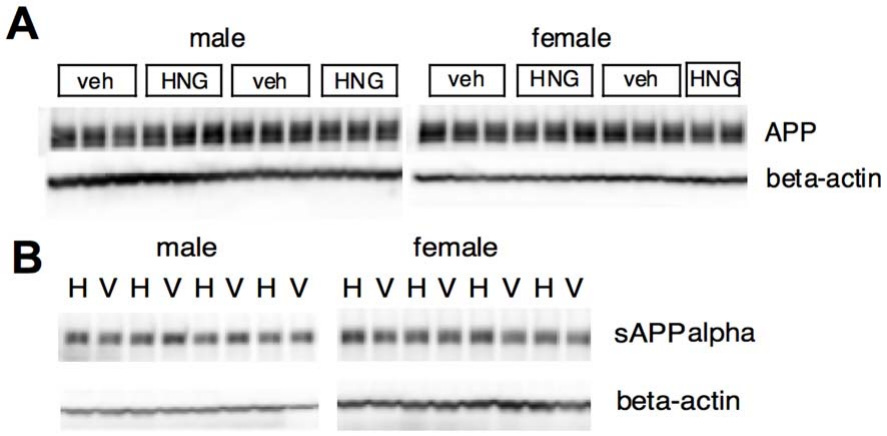
S14G-HN does not affect APP production and processing. Brain homogenate of 3xTg-AD mice treated with S14G-HN (HNG or H) or vehicle (veh or V) was subjected to immunoblot analysis using anti-APP C-terminus (**A**) or anti-sAPPalpha (**B**) (upper panels) and anti-beta-actin (lower panels) antibodies.

To understand how S14G-HN suppresses Abeta accumulation, we next examined the levels of full-length APP and sAPPalpha, a cleaved product of alpha-secretase. We performed immunoblot analysis of brain homogenate using antibodies that specifically recognize C-terminal regions of APP and sAPPalpha, respectively (Fig. 5). Quantitative analysis showed no significant difference in levels of full-length APP and sAPPalpha between S14G-HN and vehicle treated mice (number of animals; vehicle male = 7, female = 9, S14G-HN male = 9, female = 7): relative intensity of full-length APP and sAPPalpha bands in S14G-HN-treated brain homogenate (indicated as percentage of corresponding vehicle control, mean±SD) was 98±17 (male), 106±15 (female) and 81±15 (male), 98±16 (female), respectively. In addition, we detected no difference in sAPPbeta level between S14G-HN and vehicle control (data not shown). These results suggest that S14G-HN altered neither APP production nor processing by secretases.

### S14G-HN increased neprilysin expression and activity

**Figure 6:**
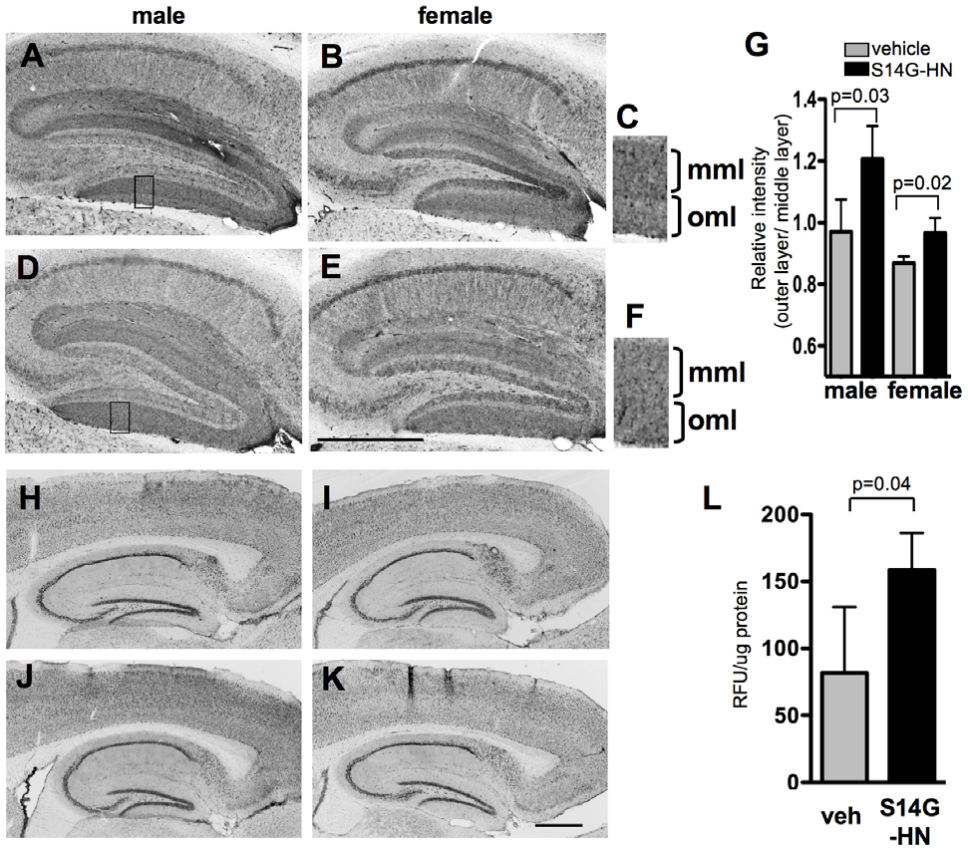
Effect of S14G-HN on NEP and IDE. **A–F, H–K.** Brain sections were subjected to immunostaining using anti-NEP antibody (**A–F**) or anti-IDE (**H–K**) and biotin-conjugated secondary antibody followed by visualization with ABC method. **A, B, H, I.** vehicle-treated mice, **D, E, J, K.** S14G-HN-treated mice. **C, F.** magnified view of boxed region in A and D, respectively. Bars  = 500 µm. mml: middle molecular layer, oml: outer molecular layer. **G.** Result of quantitative analysis on NEP is shown as mean±SD. P =  indicates p value of t-test. **L**. F11 cells were treated with S14G-HN or vehicle for 2 hours. Specific NEP activity in cell lysate was calculated by subtracting activity cleaving DAGNPG substrate in the presence of NEP inhibitor, phosphoramidon, from the total activity and shown in mean±SD (n = 4) of relative fluorescent units (RFU)/µg protein.

We next examined expression of Abeta-degrading endopeptidase, neprilysin (NEP), in S14G-HN and vehicle treated mouse brains (Fig. 6A–F). NEP level is reduced in the specific regions of brain, such as hippocampus, with AD and aging [47], [52]. In the dentate gyrus, lower NEP expression was observed in the outer molecular layer but not in the middle molecular layer by aging [47]. We performed immunohistochemical staining of brain sections using anti-NEP antibody (Fig. 6A–F). To evaluate the staining results quantitatively, we measured staining intensity of the middle and outer molecular layers. The raw values of measured staining intensity of outer molecular layer of vehicle and S14G-HN-treated mice (arbitrary units, mean±SD) were 38.2±12.0 (male), 37.5±6.4 (female) and 40.5±7.0 (male), 38.6±4.8 (female), respectively. To normalize the variation in overall staining intensity of sections, the ratio of staining intensity (outer layer/middle layer) was calculated. The ratio was significantly higher in S14G-HN-treated mice than control in both male (n = 3) and female (n = 4) (Fig. 6G). These results suggest that S14G-HN sustains or increases NEP expression in specific brain regions. We also performed immunohistological staining of brain sections with anti-insulin degrading enzyme (IDE), another major Abeta-degrading enzyme [53], [54] (Fig. 6H–K). Since levels of IDE in hippocampal CA2 and CA3 regions but not CA1 region are reduced in AD cases [55], we measured staining intensity of IDE in these brains. No significant difference was detected in levels of IDE in both CA1 and CA3 regions between S14G-HN and vehicle treated mice: relative intensities of IDE in S14G-HN-treated brain homogenate (indicated as percentage of corresponding vehicle control, mean±SD, n = 3) were 103.5±7.0 (male), 95.0±12.3 (female) in CA1 region and 91.4±6.9 (male), 104.4±3.1 (female) in CA3 region.

We further examined the effect of S14G-HN on NEP activity using neuronal F11 cell line. After 2 hours of treatment of cells with S14G-HN, we measured NEP activity with Dansyl-D-Ala-Gly-4-nitro-Phe-Gly-OH (DAGPG) in the presence or absence of phosphoramidon, a NEP inhibitor (Fig. 6L). Specific NEP activity in cells treated with S14G-HN was significantly increased, suggesting that S14G-HN can modulate NEP activity in neurons.

### S14G-HN did not affect tau phosphorylation in 3xTg-AD mice

**Figure 7:**
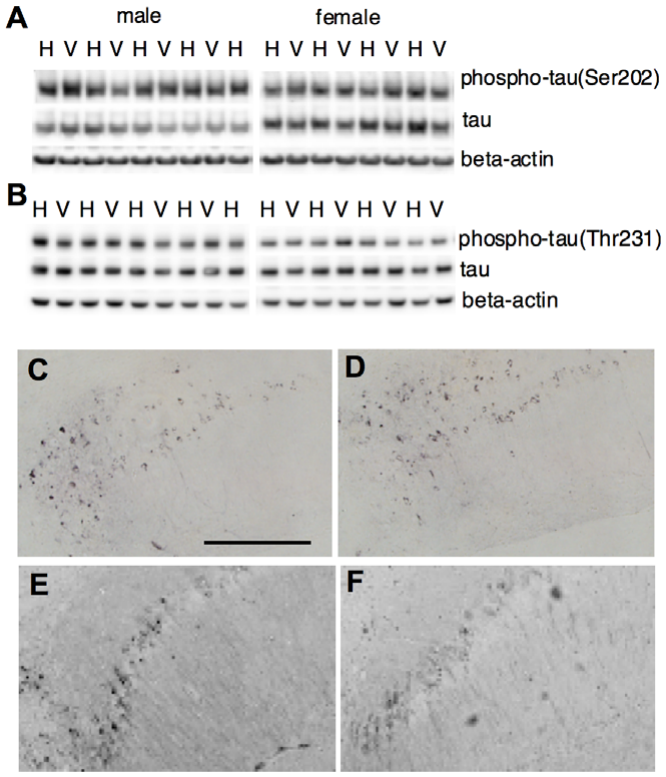
Effect of S14G-HN on tau phosphorylation. **A, B.** Brain homogenate of 3xTg-AD mice treated with S14G-HN (H) or vehicle (V) was subjected to immunoblot analysis using anti-phosphorylated tau (Ser202) (AT8) (upper panel in A), anti-phosphorylated tau (Thr231) (AT180) (upper panel in B), anti-total tau (tau46) (middle panels) and anti-beta-actin (lower panels) antibodies. **C–F.** Brain sections were subjected to immunostaining using anti-phosphorylated tau (Ser202) (AT8) antibody (**C, D**) or anti-phosphorylated tau (Thr231) (AT180) antibody (**E, F**) and biotin-conjugated secondary antibody followed by visualization with ABC method. Representative images of hippocampal CA1 region of vehicle (**C, E**) and S14G-HN (**D, F**) treated mouse sections are shown. Bar  = 200 µm.

A major characteristic of 3xTg-AD mice is age-dependent progression of tau pathology. We thus examined the effect of S14G-HN on tau pathology. Phosphorylation of tau protein was assessed by immunoblot using phosphorylation site-specific antibodies (AT8, AT180, and AT270 for Ser208, Thr231, and Thr181, respectively). Total tau was assessed by tau46 antibody. The level of total tau (normalized by actin) was not changed by S14G-HN (Fig. 7A,B). The relative intensity of bands in S14G-HN-treated mouse brain homogenate (percentage of corresponding vehicle control, mean±SD) was 100±21 (male) and 112±17 (female). The ratio of phosphorylated tau (Ser202) to total tau was not different between S14G-HN treated and control mice: the value of S14G-HN calculated as percentage of corresponding vehicle control (mean±SD) was 101±39 (male) and 95±23 (female). No significant difference between S14G-HN treated and control mice was detected in phosphorylation of Thr231 (Fig. 7B) and Thr181 (data not shown). Consistently, no difference was observed between vehicle and S14G-HN treated mouse brains by immunohistochemical analysis using anti-phosphorylated tau antibody AT8 (Fig. 7C, D) and AT180 (Fig.7E, F). Thus, S14G-HN showed no effect on tau pathology at least at the early plaque-bearing stage.

### Neuroprotective effect of S14G-HN in 3xTg-AD mice

**Figure 8:**
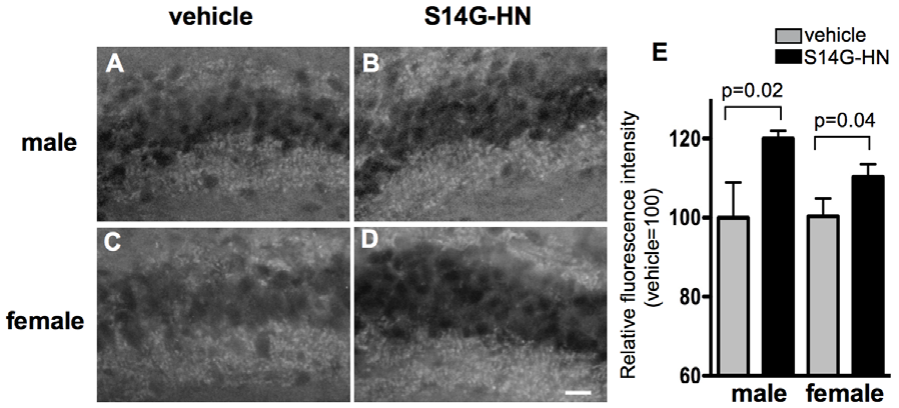
Effect of S14G-HN on synaptic degeneration. **A–D.** Brain sections were subjected to immunostaining using anti-synaptophysin antibody followed by visualization with avidin-fluorescein. Representative images of hippocampal CA3 region of vehicle (**A, C**) and S14G-HN (**B, D**) treated mouse sections are shown. Bar  = 20 µm **E.** Synaptophysin fluorescence intensity in 25 µmX80 µm area of the hippocampal CA3 region was measured. The relative value of S14G-HN-treated group (n = 3) to vehicle group (n = 3) is shown as mean±SD. P =  indicates p value of t-test.

To evaluate the effect of S14G-HN on neurodegeneration in 3xTg-AD mice, we examined synaptic density by detecting a synaptic marker synaptophysin (Fig. 8A–D). Compared with the vehicle control, the levels of synaptophysin in hippocampal CA3 region of S14G-HN mice were significantly higher in both male and female mice (Fig. 8E); average of arbitrary units (mean±SD) were 44.8±3.9 (vehicle), 53.8±0.7 (S14G-HN) in male mice and 23.7±1.0 (vehicle), 26.2±0.8 (S14G-HN) in female mice. There results suggest a protective effect of S14G-HN against synaptic degeneration.

**Figure 9:**
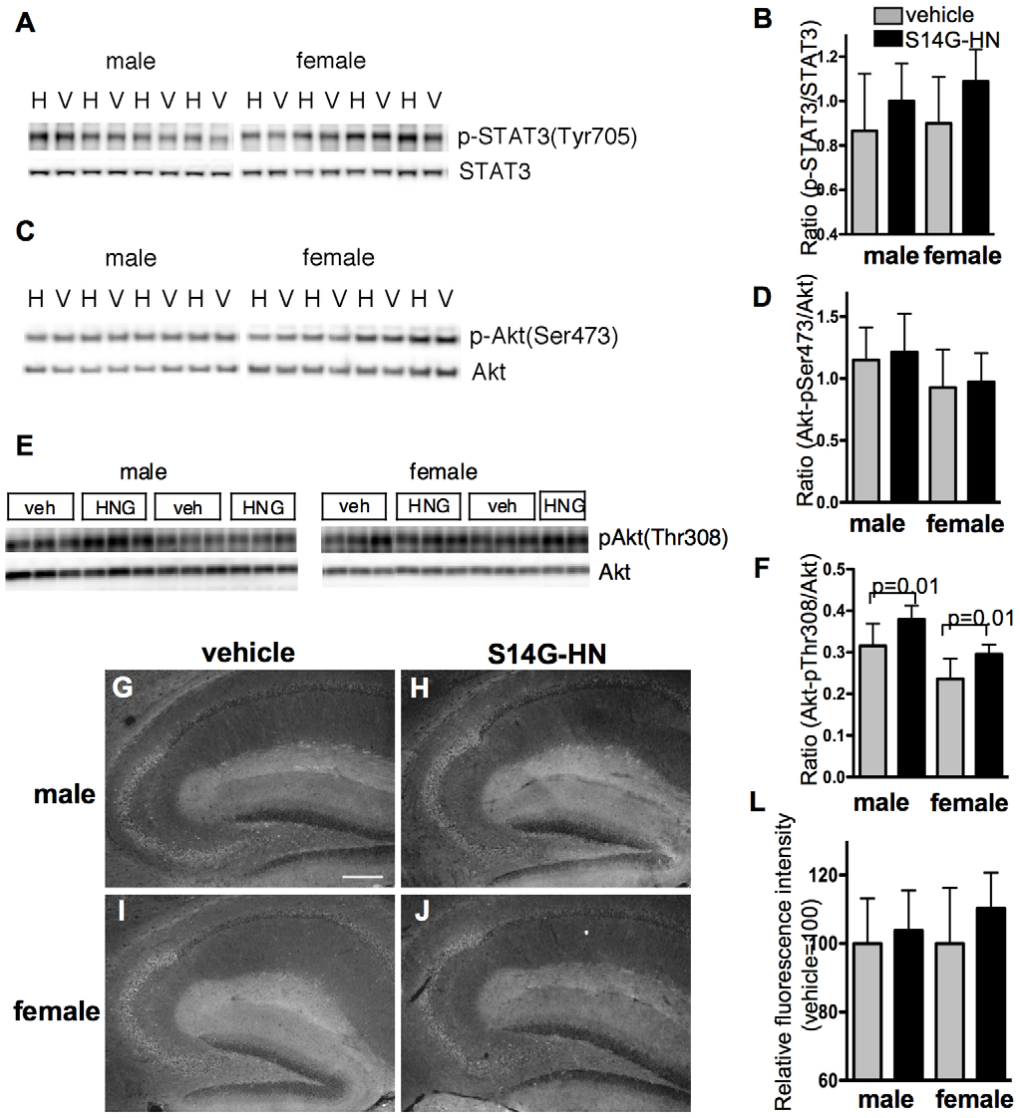
Effect of S14G-HN on activation of signaling molecules. **A, C, E.** Immunoblot analysis of phosphorylation of STAT3 (A) and Akt (C,E). Brain homogenate of 3xTg-AD mice treated with S14G-HN (H or HNG) or vehicle (V or veh) was subjected to immunoblot analysis to detect phosphorylated proteins (upper panels) and total proteins (lower panels). **B, D, F.** Quantitative analysis of immunoblots. Intensity of bands represented in A, C, E were measured (number of animals; vehicle male = 7, female = 9, S14G-HN male = 9, female = 7) and the ratio of phosphorylated proten/total protein are shown as mean±SD. P =  indicates p value of t-test. **G–J.** Brain sections were subjected to immunostaining using anti-phospho-Tyr705 STAT3 and anti-rabbit-DyLight488 antibodies. Representative images of hippocampal region of vehicle (**G, I**) and S14G-HN (**H, J**) treated mouse sections are shown. Bar  = 200 µm. **L.** Quantitative analysis of immunohistochemistry. The fluorescence intensity of phosphorylated STAT3 in 75 µmX200 µm area of the hippocampal CA3 region was measured. The relative value of S14G-HN-treated group (n = 3) to vehicle group (n = 3) is shown as mean±SD. P =  indicates p value of t-test.

Since STAT3 and Akt have been implicated in neuroprotective activity of HN [26], [56], we examined activation of STAT3 and Akt in 3xTg-AD mouse brains by immunoblot analysis of total brain homogenate (Fig. 9A,C,E). The ratio of phosphorylated STAT3 (Tyr705) to total STAT3 showed a weak trend toward increase in STAT3 activity in S14G-HN-treated mice compared with control mice, but no significant difference was detected (Fig. 9B). In the immunohistochemical analysis using anti-phospho-STAT3 antibody, both overall staining (Fig. 9G–J) and quantitative measurement of staining intensity in CA3 region (Fig. 9L) showed no significant difference between S14G-HN-treated and control mouse brains. As for Akt activation, the phosphorylation level of Ser473 showed no difference by the treatment (Fig. 9C,D), whereas phosphorylation of Thr308 was significantly higher in S14G-HN-trated mouse brains than control (Fig. 9E,F). These results can be interpreted that S14G-HN might induce transient activation of STAT3 and Akt signaling cascades but may not cause continuous activation of signaling molecules *in vivo*.

## Discussion

This study demonstrated that 3-month treatment of a highly potent HN derivative S14G-HN suppressed the deterioration of cognitive and memory functions (Fig. 3), synaptic degeneration (Fig. 8) and the increase in amyloid burden (Fig. 4, Table 1) in 3xTg-AD mice. Since Abeta, particularly soluble Abeta oligomers, have been demonstrated to induce synaptic dysfunction and memory impairment [57], the improved cognitive function by S14G-HN may be at least partly attributed to the reduced Abeta level in S14G-HN-treated mouse brains. The pro-cholinergic effect of HN might also contribute to the S14G-HN-induced cognitive benefit, since HN ameliorates amnesia caused by muscarinic receptor antagonists [21], [22], [23]. Although reduced Abeta level in brains of S14G-HN-treated mice was confirmed by both ELISA and immunohistological analysis (Fig. 4, Table 1), no significant change was observed in levels of full-length APP, sAPPalpha, and sAPPbeta in whole brain homogenates of these mice (Fig. 5). This indicates that S14G-HN does not modulate APP production or secretase activities *in vivo*. On the other hand, we observed higher levels of NEP in the outer molecular layer of hippocampal formation in S14G-HN-treated mice than in control mice (Fig. 6A–G), while IDE levels were not affected by S14G-HN treatment (Fig. 6H–K). NEP activity was also elevated by S14G-HN treatment *in vitro* (Fig. 6L). These observations suggest that the higher level of NEP in some brain regions contributes to the reduced Abeta level in brains of S14G-HN-treated mice. The molecular layer of dentate gyrus comprises the dendrites and axons arising from the entorhinal cortex and the intrinsic systems [58], indicating this region is susceptible to Abeta toxicity. In fact, soluble Abeta interfered with long-term potentiation in CA1 and dentate gyrus of the hippocampus [59], [60] and spine density is decreased in the outer layer of the dentate gyrus of AD mouse models [61], [62]. Therefore, the reduction of Abeta level in the molecular layer through increase in local NEP levels may contribute to S14G-HN-dependent amelioration of memory impairment in 3xTg-AD mice.

A behavioral test demonstrated that S14G-HN rescued cognitive function in 3xTg-AD male mice, whereas it showed a less clear effect in female mice (Fig. 3). The difference in HN's effect between genders may be attributed to the difference in the stage of Abeta pathology, because 3xTg-AD female mice showed more aggressive Abeta pathology than male mice in the plaque-bearing stage (Fig. 4) [37]. Namely, S14G-HN can induce high enough NEP levels to reduce Abeta level for preserving cognitive function in the early Abeta accumulating stage, while it was not enough in the advanced plaque-bearing stage.

HN-like molecule was detected in non-CNS organs [17], [27], [46], and the level of HN in serum was decreased age-dependently in human and rodents [27]. Given that the systemic administration of S14G-HN showed an effect similar to that of intracerebroventricular injection of S14G-HN [22], [25], it is hypothesized that HN circulated in blood stream is transferred into brain by a so far unidentified mechanism [4], and that serum level of HN correlates to the level and effectiveness of HN in brain. It is interesting to note that the NEP level in outer molecular layer is decreased by aging [47]. Taken together with our finding of NEP levels in outer molecular layer of hippocampal formation (Fig. 6), age-dependent decrease in endogenous HN levels associated with low NEP expression may be linked to increased risk for progression of AD by aging.

This study showed that both total amount and phosphorylation status of tau were unaffected by S14G-HN treatment in 3xTg-AD mice (Fig. 7), suggesting that HN has no effect on tau pathology. In 3xTg-AD mice, tau pathology becomes apparent between 12 to 15 months of age and staining with PHF1 antibody, a marker of late stage of tau pathology, is evident at 18 months of age [36]. No significant gender difference was observed for onset and progression of tau pathology [37]. The cognitive decline was reversed by Abeta immunotherapy in young 3xTg-AD mice [63], indicating that the reduction of soluble Abeta level is sufficient for the prevention of memory impairment in the early stage of Abeta pathology. However, in aged 3xTg-AD mice with advanced Abeta and tau pathologies, reduction of soluble Abeta alone did not improve the cognitive phenotype, while reduction of both soluble Abeta and soluble tau ameliorated cognitive deficit [64]. We performed behavioral tests with these mice at 16 to 17 months of age, which corresponds to mid-stage of tau pathology. It is thus possible that progressing tau pathology contributed to cognitive decline in these mice, and that the lack in the effect of S14G-HN on tau pathology may be a reason for the lack in significant effect of S14G-HN on behavioral tests in female mice, which carry advanced Abeta burden [37]. On the other hand, in the transgenic mice with human P301S mutant tau, synaptic dysfunction and its loss were detected prior to the appearance of fibrillary tau, indicating that soluble tau causes these manifestations [65]. In our study, S14G-HN-treated female mice showed potentially better cognitive functions, though not statistically significant, compared to the vehicle-treated male mice despite of the similar levels of Abeta in these two groups of animals (Figs. 3 C,D, 4A). If soluble tau contributes to the cognitive impairment of 3xTg-AD mice at this pathological stage, S14G-HN might have certain antagonistic effects on soluble tau-induced cognitive dysfunction in addition to that induced by Abeta. Alternative interpretation is that systemic effects of S14G-HN may contribute to the improvement of cognitive functions. Halagappa et al. [66] showed that intermittent fasting improved cognitive functions of 3xTg-AD mice without reducing phospho-tau levels and proposed that dietary manipulation increases the resistance of neurons to the adverse effects of tau pathology. HN injected in brains of rats modulates glucose metabolism by improving hepatic and peripheral insulin sensitivity [27]. It is, thus, possible that S14G-HN might have similar systemic effects as dietary manipulation. Moreover, several studies suggest that insulin resistance may increase the risk of AD through multiple mechanisms including increases in Abeta and inflammation [67], [68]. Therefore, an action mechanism of HN that ameliorates insulin resistance and hyperglycemia [27] may contribute to beneficial effects of HN on AD-related pathologies.

Previous studies demonstrated that several signaling molecules are involved in HN's activities *in vivo* and *in vitro*. STAT3 and related kinase(s) are involved in the anti-neuronal death activities of HN *in vitro* [14], [56] and activated *in vivo* [27], [69]. In addition to anti-cell death effects, these kinases are also involved in the antagonizing effect of HN against amnesia caused by muscarinic receptor blockade *in vivo* [23]. STAT3 contributes to the effect of HN on peripheral insulin action [27], [28]. ERK and PI3K-Akt pathways are implicated in neuroprotection of HN against cerebral ischemia/reperfusion injury *in vivo* [25], [26]. p38 and STAT3 are involved in apoptosis induced by trophic factor withdrawal in human leukemia cell line and pancreatic beta-cell line, respectively [28], [70]. In this study, we did not detect significant changes in phosphorylation of Tyr705 in STAT3 and Ser473 in Akt, while phosphorylation of Thr308 in Akt was significantly increased in S14G-HN-treaed 3xTg-AD mouse brains compared with control (Fig. 9). Since full activation of Akt requires dual phosphorylation on both Ser473 and Thr308 [71], these findings are insufficient to prove the involvement of PI3K-Akt pathway in HN-mediated neuroprotection in 3xTg-AD mice. Considering that PDK1 (phosphoinositide-dependent protein kinase-1), a major kinase for phosphorylation of Thr308 in Akt, plays an indispensable role in Akt-independent cell survival signaling pathway induced by brain-derived neurotrophic factor [72], it is speculated that S14G-HN may activate the PDK1-mediated neuroprotective mechanism. In contrast, lack of increased STAT3 activation in S14G-HN-treated mouse brains (Fig. 9) is inconsistent with previous findings that STAT3 has an essential role in the neuroprotecitve effect of HN [56]. A possible reason for this discrepancy is that S14G-HN-induced activation of STAT3 (and Akt) was transient and thus we could not detect them, since we sacrificed mice 24 hours after the last treatment.

In this study, we observed that S14G-HN increased region-specific NEP levels (Fig. 6). NEP expression is regulated by APP C-terminal fragment, AICD [73]. Since S14G-HN did not change APP production and processing (Fig. 5), S14G-HN may not affect this mechanism, unless region/cell-specific effect exists. Rather, HN may evoke other unidentified mechanisms to modulate NEP expression. In fact, since NEP levels are substantially reduced but not diminished in presenilin-deficient mice, it is hypothesized that there is an AICD-independent mechanism [73]. On the other hand, we also observed that S14G-HN increased NEP activity *in vitro* (Fig. 6L). NEP activity in primary neurons was unaffected by treatment with MAPK inhibitor as well as stimulation with insulin and IL-6, which are related to Akt and STAT3 pathways, respectively [74], suggesting that these signaling pathways do not play essential roles in modulation of NEP activity. Somatostatin is a NEP modulator and enhances NEP activity via activation of somatostatin receptor coupled with G-protein, Gi/o [74]. Since HN receptor FPRL1 is coupled with Gi/o, downstream signaling cascade(s) of this receptor may play an important role in NEP regulation. Further detailed studies will be necessary to understand signaling events evoked by S14G-HN in 3xTg-AD mice, especially pathways/molecules involved in the Abeta-lowering effect.

Regarding AD-linked pathological features, previous studies revealed that HN suppresses Abeta-induced neuronal death *in vitro* and ameliorated amnesia caused by Abeta and muscarinic receptor antagonists *in vivo*. In addition to these functions, this study showed S14G-HN decreased Abeta burden in 3xTg-AD mice, which is an additional *in vivo* function of HN as a countermeasure against AD. We also showed that decreased Abeta levels were associated with increased NEP levels in hippocampal formation of 3xTg-AD mouse brains, suggesting that HN is a NEP modulator.

Considering that intranasal administration is practically feasible for clinical application, we chose intranasal treatment over systemic methods in this study. Since the lack of significant findings in some experiments, for instance memory retention in Novel object recognition task (Fig. 2D), might be attributed to suboptimal dosing, further studies will be necessary for pharmacological development. However, the present study contributes to the preclinical evidence that intranasal administration of HN represents a potential symptomatic and disease-modifying intervention in AD. We note that the intranasal administration route has been shown to provide effective delivery of peptides to the central nervous system in humans. Further, a recent preliminary study demonstrated cognitive benefits with intranasal administration of another peptide, AL-108 [75]. Additional preclinical mechanistic and toxicological studies of HN are thus warranted, and could lead to clinical studies of this peptide in AD.
